# Consumer acceptance of upcycled craft beer: a New Zealand case study

**DOI:** 10.3389/fnut.2023.1235137

**Published:** 2023-11-09

**Authors:** Francesca Goodman-Smith, Siddharth Bhatt, Simona Grasso, Jonathan Deutsch, Miranda Mirosa

**Affiliations:** ^1^Fight Food Waste Cooperative Research Centre, The University of Adelaide, Urrbrae, SA, Australia; ^2^School of Business Administration, Pennsylvania State University at Harrisburg, Middletown, PA, United States; ^3^School of Agriculture and Food Science, University College Dublin, Dublin, Ireland; ^4^College of Nursing and Health Professions, Drexel University, Philadelphia, PA, United States; ^5^Department of Food Science, University of Otago, Dunedin, New Zealand

**Keywords:** food waste, upcycled food, consumer acceptance, food marketing, sustainable food

## Abstract

Upcycled foods are created from surplus food, edible food waste and by-products. Food and beverage brands are launching upcycled foods and promoting their product to consumers. Little is known about how consumers respond to upcycled foods, nor how these products can be most effectively promoted. To better understand marketing strategies for upcycled foods, two studies were conducted, one online (*n* = 300) and one in retail stores (*n* = 65), in New Zealand to examine differences in consumer sentiment toward upcycled beer. In both studies, environmental benefits were identified as the most important benefits of upcycled foods and information provision on pack and online were important promotional strategies. Consumers reported greater awareness and acceptance of upcycled food in-store, yet raised concerns relating to taste and price. Only 31% of participants surveyed in-store associated ‘no negatives’ with upcycled foods compared with 47% of consumers surveyed online. These findings help differentiate a potential promotion strategy for upcycled foods.

## 1. Introduction

Food waste is a significant issue, causing negative impacts on both the environment and the economy. Across the globe, $1 trillion of food is wasted every year (1). Twenty-eight percent of agricultural land is used to grow food that is never eaten (2), and in New Zealand, approximately 571,000 tons of food go to landfill each year (3). This food, produced for human consumption, if diverted at the right time and in the right way, could be retained in the food supply chain. Given that food waste is a major global problem, many solutions to this problem have been proposed. While an extensive review of all the solutions to food waste is beyond the scope of this research, we focus on a relatively new but promising solution, i.e., upcycling.

Upcycling is a partial solution to the global food waste problem, and a group known as the Upcycled Food Association (UFA) established in October 2019 is working to co-ordinate the upcycled food sector by defining upcycled food products and designing a certification for these products. The definition for upcycled foods proposed by the UFA is: “Upcycled foods use ingredients that otherwise would not have gone to human consumption, are procured and produced using verifiable supply chains, and have a positive impact on the environment” (2). The predicted potential value of the upcycled food industry is estimated to be $46.7 billion and has an expected compound annual growth rate of 5% over the next 6 years (4). This demonstrates that while food upcycling can be positive economically, the sector also has the potential to create jobs and focus on the production of healthy foods (5), offering customers more sustainable food choices. Further, there is an emerging body of literature that investigates the commercial potential of upcycled foods (6–18). This stream of research has inquired into many aspects of consumer acceptance of upcycled foods. These aspects range from consumers’ awareness about upcycled foods to their willingness to pay for such foods. These findings suggest a preliminary acceptance of upcycled foods by consumers and point to the need for more research on this topic.

The current research is inspired in part by the promise of upcycled foods to alleviate the food waste problem and in part by the findings indicating consumer interest in sustainable food consumption. While the existing literature on upcycled foods is encouraging (5–21), equally noteworthy is research on consumer perceptions of how their food consumption impacts the environment. The International Food Information Council (IFIC) survey in 2021 found that 42% of participants believe their food and beverage purchasing decisions can (moderately (~24%) or significantly (~18%)) impact the environment, and amongst participants who believe their decisions have a significant impact on the environment, two in three recognize the environment as a key purchase driver.^1^ Purchasing from businesses and companies that have aligning value sets, especially concerning action to mitigate environmental and social issues is a mechanism for consumers to make their consumption more sustainable (22). The Colmar Burton Better Futures Survey for 2020 showed that 70% of New Zealanders are looking for claims/labels to help them identify more sustainable choices and 67% want to make eco-conscious decisions, even if they cost more (23), and the IFIC 2021 survey found that for 53% of participants, a greater understanding of the impact of their purchases would influence their purchasing decisions (see text footnote 1).

Given that consumers are sensitive toward the environmental impact of their consumption, it is not surprising that the food and beverage industry is responding to this trend. Food and beverage manufacturers are exploring opportunities to offer more sustainable products to consumers that reduce food waste and associated emissions by upcycling food waste into ingredients and finished foods. One such company is Citizen Collective, based in New Zealand. Citizen Collective uses surplus bread to produce craft beer, and the spent grain from making beer is then utilized to create artisan bread, thus creating a closed-loop system. To produce the beer, surplus bread is processed into croutons which replace 25% of the malt and the fermentable starch is extracted from the croutons. The flour for the artisan bread is created by drying and milling the bread-brew mash and replaces 15% of the regular flour in the loaves. Bread is the most wasted food by New Zealand households, costing New Zealanders NZD 62.5 million annually and amounting to 9.6% of all ‘avoidable’ food waste at a household level (24). Upcycling provides a solution for consumers to help reduce food waste by altering their purchasing decisions. While the effort by Citizen Collective is in the right direction, the commercial success of their product(s) is largely dependent on how well consumers accept such non-traditional products.

Only 10% of consumers surveyed in a recent New Zealand study reported awareness of upcycling (19). Aschemann-Witzel and Peschel (20) suggest that it is important to “pre-test” new food products, including upcycled food products via soft launches and in-store tastings. Grasso and Asioli (14) also recommend that research be carried out using real products in the field (i.e., in supermarkets). Most consumer research on upcycled food to date has been conducted using online survey only (10, 12, 14–19, 25) yet in-store surveys present the advantage of having someone there not only to promote the product but also to discuss the concept of upcycled foods with consumers (20).

This study sought to inform effective promotional strategies for upcycled craft beer by examining differences in consumer sentiment toward upcycled craft beer when the product was presented in the retail store compared to an online environment. While the studies were conducted with upcycled craft beer and are exploratory in nature, several of the findings can be generalized across upcycled food and beverages of different types.

## 2. Methods

Given the nascent state of literature on this topic, there was a need to employ exploratory research methods. The researchers used non-probabilistic sampling and an exploratory lens in order to uncover some basic insights as to consumers’ acceptance of upcycled craft beer. Accordingly, a survey with both open-ended and closed- ended questions was developed. The initial survey was developed by the researchers in collaboration with Citizen Collective and pre-tested with customers in three retail stores on the 22nd and 23rd of January 2021. The survey was refined as a result of participant and researcher feedback. These changes reduced the average response time from four-and-a-half minutes to one-and-a-half minutes. The final survey was then administered via two mechanisms (1) in-store and (2) online, to understand consumer sentiment toward upcycled craft beer. As noted above, executing the survey both online and in-store allowed for triangulation of results and improved confidence in the findings. Next, we describe the process of data collection in both the online and in-store environments before reporting the results.

### 2.1. In-store survey

In-store sampling and surveying introduced consumers to upcycled beer at seven full-scale retail stores (New World and PAK’nSAVE) operated by a large New Zealand retailer Foodstuffs. Research was undertaken in two major cities (Auckland and Wellington, the two cities with the greatest proportion of stores selling upcycled craft beer) in February 2021. The researchers worked with the Merchandise Management team at Foodstuffs to recruit stores as research sites in these locations.

To provide the customers with a true in-store experience, Citizen staff conducted the sampling and discussed the product with customers and a researcher accompanied the Citizen team to administer the survey. A tasting station was set up in the craft beer section of the store and the research team invited shoppers who entered the craft beer area and sampled the beer to participate in the survey. Prior to offering samples photo identification was sought to determine that all participants were aged over eighteen and legally allowed to be served alcohol. During product tasting, Citizen staff educated the customer about the product, including a verbal summary of the product manufacturing process. This summary informed customers that the craft beer was made from surplus bread sourced from supermarkets, and the spent grain from the beer-making process was converted back into artisan bread; a process known as upcycling. Customers who sampled the beer were then invited to complete a short survey. The survey included six questions covering general selection criteria for craft beer, prior awareness of upcycled foods, perceived benefits and concerns associated with upcycled foods, appeal of upcycled craft beer compared to conventional craft beer and factors that would aid future purchasing decisions concerning upcycled foods (see Supplementary Table 1). In order to emulate a true in-store sampling experience, of which a core component is hearing about the story of the product from the producer and what makes this product different from others on the market, customers were informed of the upcycled nature of the product prior to undertaking the survey. To overcome potential bias when gauging consumer awareness of upcycled foods in the survey the question was worded “Have you ever heard of upcycled foods before today?” The temporal reference in this question was included in order to focus the respondent on prior awareness. These conditions were replicated in the online survey format in the form of a contextual paragraph about the product at the beginning of the survey (see Section 2.2).

The survey took approximately one-and-a-half minutes to complete, and shoppers were free to decline to answer any questions in the survey. Sixty-five shoppers participated in the in-store survey (*n* = 65). Ethics approval was gained from the University of Otago Ethics Committee (Ref No: 20-09B) in December 2020. Included in the approval of the Ethics application were the survey questions, survey protocol, Participant Consent Form and Participant Information Sheet.

### 2.2. Online survey

The survey was then replicated online in March 2021 using an online platform called Yabble and was conducted according to the code of ethics for the Research Association of New Zealand. In March 2021, PAK’nSAVE and New World panels were used to administer the same survey to craft beer consumers. Shoppers who did not purchase craft beer were excluded through a vetting question at the beginning of the survey “*Do you purchase craft beer from New World or PAK’nSAVE supermarkets*?” If respondents answered ‘yes’ they were able to proceed to the survey. If respondents answered ‘no’, the survey did not progress. Three hundred consumers answered ‘yes’ and completed the online survey (*n* = 300), participants were entered into a prize draw to win one of four $50 New World or PAK’nSAVE gift cards. While there was no live sampling, the online survey participants were provided with a short blurb and a photo of Citizen Beer before they begun the survey to mimic the information provided to participants surveyed in store:

“Citizen beer is a new craft beer that takes surplus bread from supermarkets, like New World and PAK’nSAVE and turns this into craft beer, this process is known as upcycling.”

### 2.3. Analysis

Data from the surveys were exported from Qualtrics survey software (Qualtrics, Provo, UT) and imported into Microsoft Excel. Descriptive statistics were undertaken to determine the percentage of participants in the online sample and the in-store sample that selected each multi-choice answer. A test of two proportions was then undertaken to understand whether there were statistically significant differences between responses of participants surveyed in-store and participants surveyed online. A value of *p* < 0.05 was selected as the threshold for a statistically significant difference between samples due to the small sample size.

## 3. Results

Of the in-store sample, 69% percent of participants were male. The largest proportion of participants were aged 18–34 (42% of participants), with the smallest representation in the over 65 years category (6% of participants). Of the online sample, 58% of participants were male, and the largest proportion of participants were aged 35–54 (45% of participants), with the smallest representation aged 18–34 (12 percent of participants) (Table 1).

**Table 1:** Demographic variables of participants surveyed in-store and online (*n* = 365).

	Surveyed in-store (*n* = 65) *n* (%)	Surveyed online (*n* = 300) *n* (%)
Age (years)		
18–34	27 (42)	36 (12)
35–54	23 (35)	135 (45)
55–65	11 (17)	84 (28)
>65	4 (6)	45 (15)
Gender		
Male	45 (69)	174 (58)
Female	20 (31)	126 (42)

**Figure 1:**
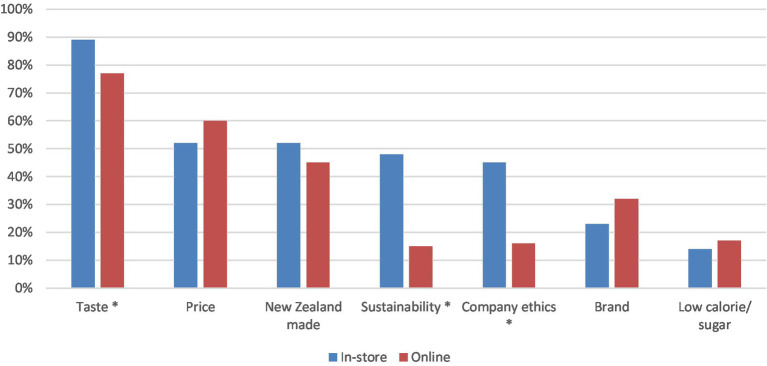
Important attributes for consumers when selecting craft beer. Asterisk shows significant differences between the in-store and online categories (*p* < 0.05).

In setting the context for the survey, participants were asked about the aspects they found important when selecting craft beer. Irrespective of whether customers were surveyed in-store or online, the most important three attributes identified were taste, price, and Country of Origin (New Zealand made). Next, inferential tests (z tests) were conducted to determine whether there were significant differences between the two samples (online vs. in-store) on key attributes of upcycled craft beer. A *z*-test revealed that significantly more participants surveyed in-store identified taste (z = 2.20, *p* < 0 0.05), sustainability (z = 5.89, *p* < 0.01), and company ethics (z = 5.13, p < 0.01) as important attributes compared to participants surveyed online (see Figure 1).

**Figure 2:**
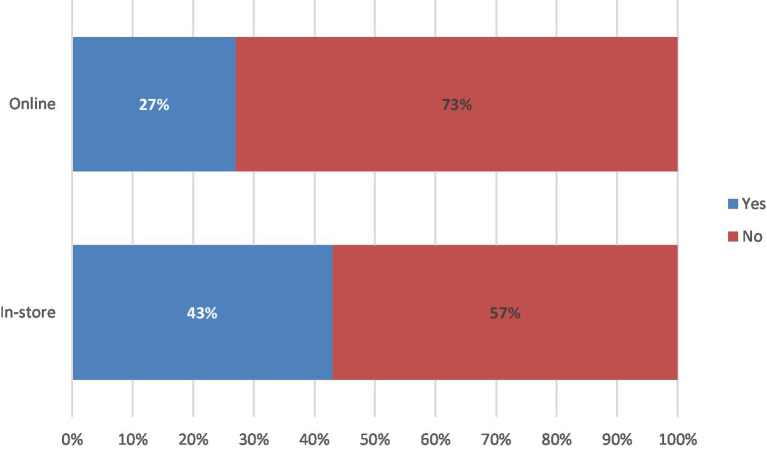
Awareness of consumers regarding the term ‘upcycled food’ before survey date.

The survey then sought to understand customer awareness for upcycled foods. Accordingly, a *z*-test was conducted to test the difference in consumer awareness of upcycled foods between the two samples (online vs. in-store). Overall, a greater proportion of participants surveyed in-store reported being aware of upcycled foods prior to participating in the survey compared to participants in the online survey (43% in-store vs. 27% online; z = 2.57; *p* < 0.05; Figure 2).

**Figure 3:**
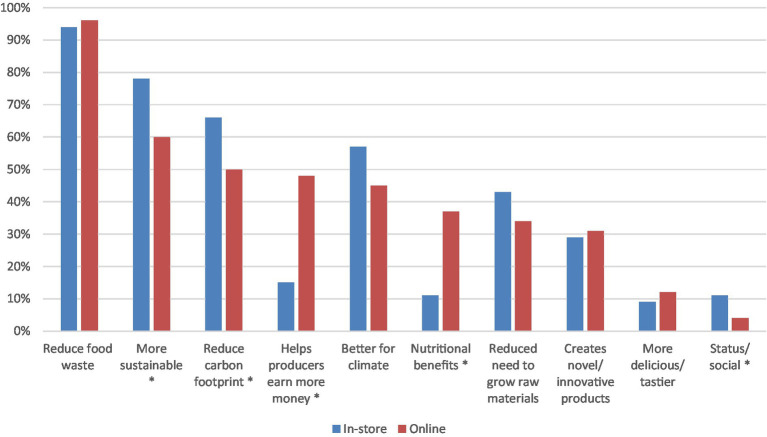
Perceived benefits that consumers associated with upcycled foods. Asterisk shows significant differences between the in-store and online categories (*p* < 0.05).

After gauging the importance of craft beer attributes and awareness of upcycling in foods, the survey proceeded to assess the benefits that customers sought from upcycled foods (see Supplementary Table 1). The top three benefits consumers associated with upcycled foods were the same whether participants were surveyed in-store or online. These benefits were the potential for upcycled foods to reduce food waste (98% in-store, 96% online), that they may be more sustainable (78% in-store, 60% online) and that they may reduce the carbon footprint of a product (66% in-store, 50% online). Again, *z*-tests were conducted to test differences between the two samples. Significantly more participants who were surveyed in-store identified the benefit of upcycled foods as being more sustainable (78%, z = 2.80, *p* < 0 0.01), that they reduce carbon footprint (66%, z = 2.37, *p* < 0.05), and their ability to increase social status (11%, z = 2.23, *p* < 0.05) compared to those surveyed online. On the other hand, significantly more participants who were surveyed online found nutritional benefits (37%, z = −4.10, *p* < 0.01) and the ability of upcycled foods to help producers earn more money (48%, z = −4.8271, *p* < 0.01) as important compared to those surveyed in-store. The proportions for all the benefit appeals are reported in Figure 3.

The survey then asked consumers to think about potential concerns associated with upcycled foods (see Figure 5). The researchers tested for differences between the two samples on this measure using *z*-tests. Of those surveyed in-store, significantly more (35%, z = 2.90, *p* < 0.01) participants were concerned as to whether the upcycled food product would taste good, compared to those surveyed online (19%). Also in-store, concern as to whether the product would be expensive was identified by 34% of participants, more so than when surveyed online (13%, z = 4.08, *p* < 0.01), and whether the product would be of good quality was a concern identified by 30% of participants. When surveyed online, 47% of participants believed there were no negatives associated with upcycled food products, significantly more than those surveyed in-store (31%) (*z* = −2.39, *p* < 0.05). Only 12% of in-store participants and 18% of online participants highlighted food safety as a potential negative associated with upcycled foods.

**Figure 4:**
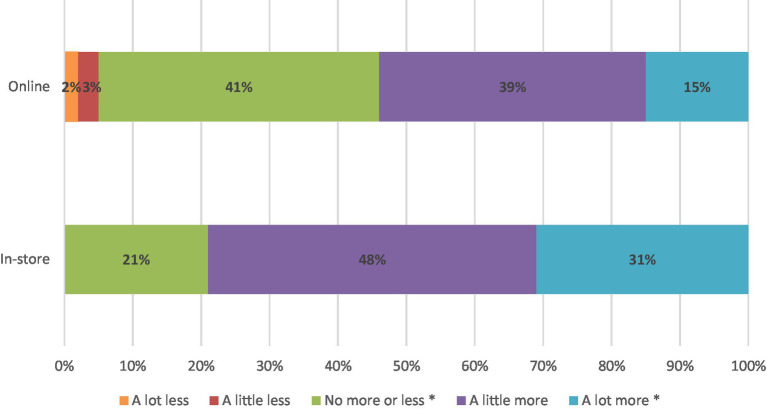
Appeal of upcycled craft beer to consumers compared with conventional craft beer.

When surveyed in-store, 79% of consumers identified that an upcycled craft beer was either ‘a little more’ or ‘a lot more appealing’ than a conventional craft beer (51/65 consumers) (see Figure 4). When surveyed online, only 54% of consumers identified that an upcycled craft beer was either ‘a little more’ or ‘a lot more appealing’ than a conventional craft beer. Like other measures, *z*-tests were conducted to assess differences between the two samples. Regarding participants who found upcycled craft beer ‘a lot more appealing’ than conventional craft beer, there was a statistically significant difference (Z = 3.01, *p* < 0.01) between respondents surveyed in-store (31%) and online (15%). Also, statistically significantly more (Z = −2.94, *p* < 0.01) participants surveyed online (41%) deemed upcycled beer as no more or less appealing, compared with 21% of those surveyed online.

It is particularly important to note that no participants identified upcycled craft beer as less appealing than conventional craft beer in the in-store survey. In the online survey, just 5% of participants identified upcycled craft beer as less appealing than conventional craft beer.

Participants surveyed in-store identified clearly highlighting on the packaging that the product is upcycled as the most important tool in encouraging uptake of upcycled craft beer, whereas, for those participants surveyed online, price was the most important enabler (see Figure 6). Price (56%, *z* = −5.49, *p* < 0.01), a dedicated upcycled area in-store (29%, z = −2.52, *p* < 0.05) and a third party upcycled endorsement (22%, z = −2.35, *p* < 0.05) were identified as important promotional tools by more of those surveyed online than in-store (see summary of results in Supplementary Table 2).

## 4. Discussion

Food waste continues to be a major challenge across the globe even when millions of people are experiencing food insecurity. Hence, research has focused on finding solutions to this pernicious problem. Of these, upcycling is a solution that tackles food waste early in the food supply chain and is therefore a better solution than composting or feeding surplus food to animals (8, 9). While intuitive, upcycling as a concept is new to the food and beverage industry. Despite their economic and environmental benefits, upcycled foods can be successful in alleviating the food waste problem only if they are widely accepted by consumers. This research was conducted to contribute to the small but growing literature on consumer acceptance of upcycled foods (6–21).

Findings from this research contain several insights for better marketing of upcycled foods. Increasing consumer awareness for upcycled products may be the first frontier. Previous studies which have attempted to understand consumer awareness of upcycled foods have asked consumers of their previous knowledge of the term ‘upcycled’ before describing the term to participants. In these studies, only 10% of New Zealand consumers surveyed in 2020 (19) and 15% of UK consumers surveyed by Grasso and Asioli in 2020 (14) reported prior awareness of upcycled foods. Studies, such as Coderoni and Perito (12, 21) which provided consumers with a description of the process of making food from by-products before asking about prior awareness of upcycled foods have found a higher awareness rate (81% in the 2019 study and 85% in the 2021 study). The present study employed the same approach, describing the process of creating beer from surplus bread to illustrate the concept before asking respondents about their prior awareness of this concept, resulting in 27% of those surveyed online and 43% of those surveyed in-store (Figure 1) reporting previous awareness of upcycled food. The increase in consumer awareness in this study and other studies which describe the concept of upcycling prior to gauging awareness compared to studies which present the term without context illustrates that overall perhaps consumers have greater awareness of the concept of upcycling, but lower awareness of the term. Therefore, producers of upcycled foods should consider presenting context when using the term upcycling to trigger recognition with consumers. Using a consistent definition, for example the definition proposed by the Upcycled Food Association (2), could be one way to achieve this, the consistency in the definition is important to reinforce the meaning of the term to consumers.

In the present study, consumers reported both greater awareness of the concept of upcycled food (Figure 2) and greater acceptance (Figure 4) in an in-store environment, compared to those surveyed online. Therefore, messaging to further raise awareness for upcycled foods may be effectively positioned in-store where overall interest in such products is heightened, and where it is possible to interact and engage with consumers. Point-of-sale messaging of suboptimal foods to increase acceptance and awareness was also identified by Aschemann-Witzel et al. (25) to be effective in communicating these foods to customers.

**Figure 5:**
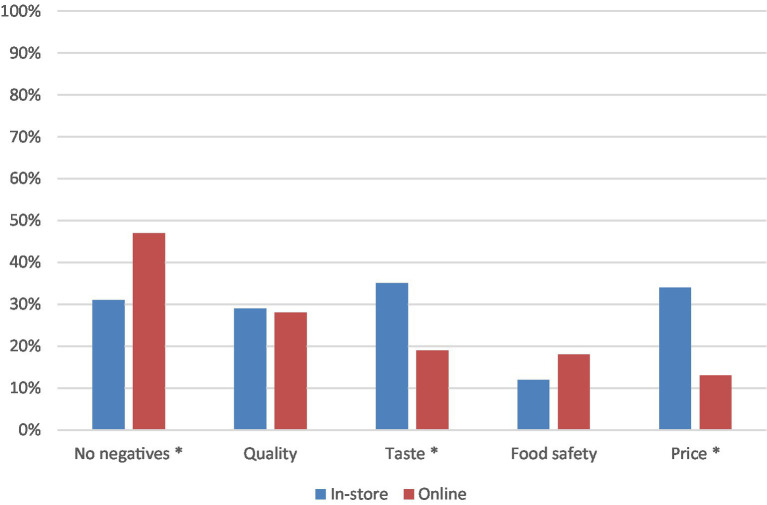
Potential negatives that consumers associated with upcycled foods. Asterisk shows significant differences between the in-store and online categories (*p* < 0.05).

**Figure 6:**
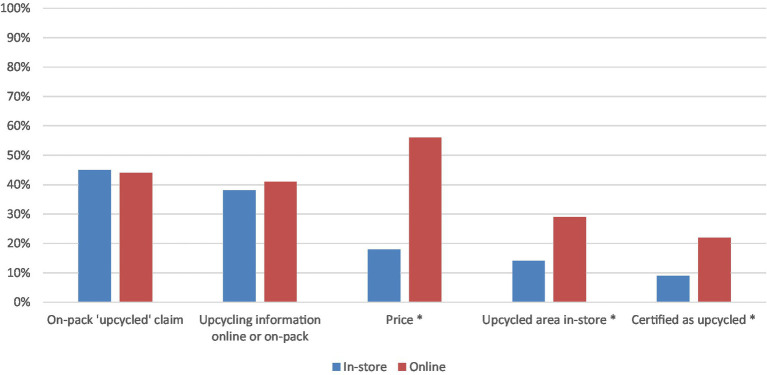
Promotional factors that would encourage consumers to purchase an upcycled craft beer over conventional craft beer. Asterisk shows significant differences between the in-store and online categories (Indicates *p* < 0.05).

A promising finding from this research was that 47% of those surveyed online and 31% surveyed in-store identified no negatives with upcycled foods (Figure 5). Of the list of possible concerns provided, concern for product quality was evident amongst those surveyed online and those surveyed in-store. Such concerns, which are natural with new products, are especially pertinent for edible products that impact one’s well-being more directly. Upcycled beer manufacturers will need to devise a strategy to assure consumers of the quality of their craft beer using appropriate marketing communications. Providing the opportunity to sample the product in-store did not appear to influence consumer concerns about quality. Awareness and assurance must be the key elements of the marketing strategy around this new product. Whether the product would taste good and whether the product would be more expensive were named as concerns by significantly more consumers surveyed in-store compared to those surveyed online. This is an interesting finding considering consumers were offered the opportunity to taste the product, and the price information was available in-store. Those surveyed online were not provided the price information nor the opportunity to taste, which may provide an explanation as to why those concerns were not top of mind. In contrast, significantly more consumers surveyed online raised price as an important promotional strategy for upcycled foods compared to those surveyed in-store (Figure 6). While the concerns need to be dealt with, there is a silver lining in the survey findings. Many consumers indicated potential benefits from consuming these products. Irrespective of whether consumers were surveyed online or in-store, benefits relating to environmental outcomes were identified as important by the greatest proportion of consumers (Figure 3); such a finding was also reported by Grasso and Asioli (14) in their case study of consumer acceptance of upcycled biscuits conducted in the UK. Positioning implications derived from careful consideration of these environmental benefits will be useful for building a promotional strategy for upcycled beer. Regardless of being surveyed in-store or online consumers identified that information on the product packaging and information online relating to the upcycled attributes and development of the product were important promotional strategies (Figure 6). Peschel and Aschemann-Witzel (17) also found that greater transparency concerning the communication of sustainability benefits of upcycled foods was associated with increased consumer likelihood to opt for an upcycled product (considered without the context of price) and that information provision about the process of upcycling can increase consumer attitudes toward these products (20). This result was particularly evident in “vice” or discretionary products, of which beer is. The present study uncovers suggestions about how the upcycled beer can be promoted (Figure 6). As with many new products, consumers are apprehensive at first due to concerns relating to quality and benefits of the new product (11). However, the role of marketing in overcoming the concerns and shaping consumer acceptance is key (11). From the results of this case study, it is evident that point-of-sale communications to build transparency about upcycled beer will likely aid in further developing consumer awareness and acceptance of the product. Promotional strategies should leverage environmental motivators and allay concerns about product quality. Comparability of pricing between upcycled products and conventional products was also a concern yet was highlighted as an opportunity that can be realized as part of a promotional strategy. While the commercial success of upcycled craft beer remains to be seen, this research provides useful directions for developing promotional strategies to increase consumer acceptance.

There were however limitations to the study, which include the unmatched demographics between the in-store survey population and the online survey population. As the demographics are not directly comparable, this should be considered when interpreting the results. The samples were also not matched by size, due to the short data collection window for in-store sampling, constrained by store availability for taste-testing sessions. It was only possible to undertake 65 surveys in-store, to increase the total sample population additional participants were recruited to participate in the online survey. Another limitation of the study was that the survey was multi-choice and therefore participants were required to choose from the answers provided which may not have represented their views accurately, as the in-store surveys were conducted on i-Pads it was decided that the addition of free text questions was not practical. There were however strengths to the study, which attempted to understand consumer sentiments to upcycled food in an in-store environment, in contrast to many consumer studies which conduct online surveys only.

## 5. Conclusion and recommendations

Four key conclusions and recommendations resulted from the study:

1. In general consumer awareness of upcycled foods should be increased and this could be done in a variety of ways, by promotion and sampling of products in-store, through claims on-pack (19), by providing information online (19), or through product certification (10, 19).
2. It is important for an agreed definition to be used so as not to confuse consumers. If we want consumers to be able to quickly recognize and value upcycled foods, the concept must be communicated clearly.
3. Future work could include sensory analysis of selected upcycled food products with consumers, before and after providing the definition of upcycled foods, to see if information provision can affect their sensory perception.
4. Future research may also provide consumers with a range of upcycled products to compare and contrast their attributes.

In the meantime, upcycled food manufacturers can use these insights to inform their future promotional strategies, which may now place increased focus on in-store product demonstrations and go forward with a reaffirmed confidence demonstrated by the positive consumer sentiments demonstrated in this New Zealand case study.

## Data availability statement

The datasets presented in this article are not readily available because restrictions apply to the availability of these data. Data were obtained from Citizen Collective and Foodstuffs NZ and are available from the authors with the permission of Citizen Collective and Foodstuffs NZ. Requests to access the datasets should be directed to francesca@fightfoodwastecrc.com.au.

## Ethics statement

The studies involving humans were approved by University of Otago Ethics Committee. The studies were conducted in accordance with the local legislation and institutional requirements. The participants provided their written informed consent to participate in this study.

## Author contributions

FG-S, MM, SB, and JD: conceptualization, methodology, and writing—original draft preparation. FG-S and MM: validation. FG-S, SB, and JD: formal analysis. FG-S: investigation, resources, data curation, project administration, and funding acquisition. FG-S, MM, SB, JD, and SG: writing—review and editing. MM and JD: supervision. All authors contributed to the article and approved the submitted version.

## Footnotes

^1^ https://foodinsight.org/2021-food-health-survey
