# Supplementary material for: Consumer acceptance of upcycled craft beer: a New Zealand case study
Source: Front Nutr. 2023 Nov 9;10:1235137. doi: 10.3389/fnut.2023.1235137 (PMC10666164; doi:10.3389/fnut.2023.1235137)
Supplement: Supplementary file 1 [file Data_Sheet_1.docx]

Supplementary Material

Consumer Acceptance of Upcycled Craft Beer: A New Zealand Case Study

**Francesca Goodman-Smith^*^, Siddharth Bhatt, Simona Grasso, Jonathan Deutsch and Miranda Mirosa**

*** Correspondence:** Francesca Goodman-Smith; francesca@fightfoodwastecrc.com.au

# Supplementary Figures and Tables

## Supplementary Table 1: Survey questions

| 1. “**Which of the following attributes are important to you when selecting craft beer?**  - *(Tick all that apply – Low calorie/ sugar; NZ made; Company ethics; Sustainability; Taste; Brand; Price).*  1. “**Have you ever heard of upcycled food before today?”** *(Yes/No)*   **Definition** of upcycled food displayed on iPad –  “Upcycled foods are food products made from ingredients that are edible and safe to eat but which would otherwise be wasted, thereby helping to prevent edible food from going to waste. For example, a soup can be made from peels, scraps, and ugly vegetables”.   1. **“In your opinion what are the benefits of upcycling food?”**  - *(Tick all that apply – Reduce food waste; Reduce carbon footprint; Nutritional benefits; Reduced need to grow raw materials; Better for the climate; More sustainable; Helps producers earn more money; Creates novel/innovative products; More delicious/ tastier; Status/ social).*  1. **“In your opinion what are the negatives of upcycling food?”**  - *(Tick all that apply – Would it taste good?; Would it be good quality?; Would it be more expensive?; Would it be safe to eat?; No negatives).*  1. **“Knowing this beer is upcycled… compared with other craft beers is it…?”**  - *(A lot more appealing; A little more appealing; No more or less appealing; A little less appealing; A lot less appealing).*  1. **“In the future what would encourage you to choose an upcycled food product over a conventional product?”**  - (*Tick all that apply – Clearly highlighting that this is an upcycled product on the packaging; Information about the upcycling process online or on the product packaging; A third party upcycled endorsement, i.e. certified upcycled; A dedicated upcycled area in-store; Price)*. |
| --- |

## Supplementary Table 2: Summary of findings

| **Awareness of upcycled foods** | **In-store (n=65)** | **Online (n=300)** |
| --- | --- | --- |
| Yes | 43% | 27% |
| No | 57% | 73% |
| **Important beer attributes** |  |  |
| Taste | 89% | 77% |
| Price | 52% | 60% |
| New Zealand Made | 52% | 45% |
| Sustainability | 48% | 15% |
| Company Ethics | 45% | 16% |
| Brand | 23% | 32% |
| Low calorie/ sugar | 14% | 17% |
| **Benefits of upcycling food** |  |  |
| Reduce food waste | 94% | 96% |
| More sustainable | 78% | 60% |
| Reduce carbon footprint | 66% | 50% |
| Helps producers earn more money | 15% | 48% |
| Better for climate | 57% | 45% |
| Nutritional benefits | 11% | 37% |
| Reduced need to grow raw materials | 43% | 34% |
| Creates novel/ innovative products | 29% | 31% |
| More delicious/ tastier | 9% | 12% |
| Status/ social | 11% | 4% |
| **Concerns around upcycled foods** |  |  |
| No negatives | 31% | 47% |
| Would it be good quality? | 29% | 28% |
| Would it tase good? | 35% | 19% |
| Would it be safe to eat? | 12% | 18% |
| Would it be more expensive? | 34% | 13% |
| **Effects of upcycling on food perceptions** |  |  |
| A lot less appealing | 0% | 2% |
| A little less appealing | 0% | 3% |
| No more or less appealing | 21% | 41% |
| A little more appealing | 48% | 39% |
| A lot more appealing | 31% | 15% |
| **Promotion suggestions** |  |  |
| Clearly highlighting that this is an upcycled product on the packaging | 45% | 44% |
| Information about the upcycling process online or on the product packaging | 38% | 41% |
| Price | 18% | 56% |
| A dedicated upcycled area in-store | 14% | 29% |
| A third party upcycled endorsement, i.e., certified upcycled | 9% | 22% |
